# Leprosy As a Complex Infection: Breakdown of the Th1 and Th2 Immune Paradigm in the Immunopathogenesis of the Disease

**DOI:** 10.3389/fimmu.2017.01635

**Published:** 2017-11-28

**Authors:** Jorge Rodrigues de Sousa, Mirian Nacagami Sotto, Juarez Antonio Simões Quaresma

**Affiliations:** ^1^Tropical Medicine Center, Federal University of Pará, Belém, Brazil; ^2^Faculty of Medicine, Department of Pathology, Sao Paulo University, São Paulo, Brazil; ^3^Center of Biological and Health Sciences, State University of Pará, Belém, Brazil

**Keywords:** *Mycobacterium leprae*, immunology, cytokines, lymphocytes, immunopathology

## Abstract

Leprosy is a chronic infectious disease whose evolution involves complex immune mechanisms of the host that influence the clinical presentation of the disease. For many years, the main interpretation of the host defense response was based on characterization of the established immune paradigm between T helper (Th) 1 and Th2 lymphocytes. However, with advances in the knowledge of immunology, new approaches have emerged along with the development of new immunological pathways that have changed the interpretation of the long-established paradigm of the polar forms of the disease, especially with the identification of new subtypes of T lymphocytes such as Th9, Th17, Th22, and Tregs. Thus, this review discusses the role of these new subtypes of T helper lymphocytes and how the development of the immune response of these cells modifies the pattern of the Th1/Th2 response in the immunopathogenesis of leprosy.

## Introduction

Leprosy is an ancient, insidious disease that causes tissue and demyelinating lesions in the peripheral nerves (1–10). Its infectious agent is *Mycobacterium leprae*, which infects macrophages, as well as dendritic and Schwann cells (11). Because of its severity, leprosy is still considered a serious public health problem (12–17). Leprosy is considered a peculiar disease given its spectral evolution. According to the Ridley and Jopling classification proposed in 1966, the interpretation model takes into account the histopathological changes associated with immunological changes. Within the spectrum of the disease, leprosy is constituted by five main clinical forms (5). The tuberculoid form represents the pole of resistance and is characterized by intense cellular immunity, with few bacilli and a limited number of lesions. The lepromatous form is at the other end of the spectrum, the susceptibility pole, in which the cellular immune response is compromised, and the skin lesions are more diffuse with intense growth of the bacillus in macrophages. The intermediate forms, borderline tuberculoid, borderline borderline, and borderline lepromatous leprosy, are immunologically dynamic, presenting oscillating characteristics between the two poles of the disease (5, 18–21).

## Immune Response in Leprosy

The first line of the interaction between *M. leprae* and the host is mediated by pattern recognition receptors that detect pathogen-associated molecular patterns (PAMPs). These recognition receptors are expressed primarily by phagocytic cells such as macrophages and dendritic cells (22–24). Previous studies have demonstrated the versatility of these receptors and the signaling cascades that can develop (25, 26). Many theories have been proposed for the immune mechanism in leprosy, which have been based on the course of the response involving the relationship between Toll-like receptors (TLRs), dendritic cells, macrophages, and lymphocytes (27, 28). Among the receptors expressed by phagocytic cells, TLR2 and TLR4 are the two major receptors involved in development of the immune response by recognition of the *M. leprae* PAMPs (29–31).

With respect to the dendritic cell response, more recent studies have identified a variety of cell subtypes that modulate the initial construction of the immune response in the polar forms of the disease (32–34). In this context, the presence of the dendritic cells of the epidermis (CD1a+) and langerin (CD207+) show particularly increased expression in the tuberculoid form. With respect to the inflammatory infiltrate, the immunostaining of dermal dendrocytes (FXIIIA+) and plasmacytoid dendritic cells (CD123+) was predominantly detected near the vessels and granulomas in the tuberculoid form (34). These findings demonstrated that the presence of dendritic cells in the epidermis or areas close to the infiltrate participate in the development of an effective immune response against *M*. leprae (34).

Macrophages are the cell population that plays a central role in the interaction between the bacillus and host. Since macrophages are the main cells that exert microbicidal activity in leprosy, many studies have already described the role of these cells in the response to cytokines, including tumor necrosis factor-alpha (TNF-α) and interferon-gamma (IFN-γ) (35, 36). In leprosy, both TNF-α and IFN-γ have been shown to bind to the cellular receptor of the macrophages, thereby changing the behavior of M0 macrophages, which undergo phenotypic modification to become M1 inflammatory macrophages. M1 macrophages produce inflammatory cytokines and enzymes such as induced nitric oxide synthase (iNOS), which induces the production of NO and consequently generates free radicals that destroy the bacillus (37–39). An alternative pathway has been identified in the lepromatous form, which is activated owing to the presence of M2 macrophages that produce anti-inflammatory cytokines (IL-4, IL-10, and IL-13), growth factors [TGF-β and basic fibroblast growth factror (FGF b)], and enzymes such as arginase 1 and IDO that contribute to the development of immunosuppressive mechanisms as well tissue repair (11, 36, 40–42). Therefore, the response of M2 macrophages plays an important role in the immunopathology of the lepromatous form of the disease, because these cells express the scavenger receptor (CD163) that may contribute to entry into the bacillus cell (11, 43).

The T and B lymphocytes play fundamental roles in the immune response since they participate in mechanisms that lead to the development of the microbicidal or humoral response in the spectrum of the disease (44–46). The two main types of T lymphocytes that are most extensively studied in the T CD4 + response pathway are the Th1 lymphocytes associated with the tuberculoid form and the Th2 lymphocytes associated with the lepromatous form (47–49). T CD8+ lymphocytes are primarily involved in the development of cytotoxicity (50, 51). Studies have shown that T CD8+ cells participate in mechanisms that lead to the destruction of the bacillus in coinfection with HIV in a reverse reaction (52, 53). In this context, by recognizing virus-infected cells, T CD8+ cells would promote the release of granzymes and perforins that destroy the coinfected cells in patients with a type-1 reverse reaction (52, 53). The response of B lymphocytes is primarily associated with the humoral response, and some studies on leprosy have demonstrated increased expression of CD20 in the tuberculoid form and borderline tuberculoid form in a reverse reaction (40, 54).

## The Th1/Th2 Paradigm in Leprosy

Leprosy represents a multifactorial complex disease model in which the bacillus modulates the behavior of the host immune response according to the pathogen’s acquired evasion mechanisms. In characterization of the immune response, the disease classically presents two clinical forms that are considered to be antagonistic, which guides understanding of the dual response pattern observed between Th1 and Th2 lymphocytes (6, 47–49, 55). In leprosy, Th1 and Th2 lymphocytes arise from the differentiation of Th0 lymphocytes, where the main cytokines involved in the process are IL-2, IL-12 (Th1), and IL-4 (Th2). Th1 cells often express CCR5 and CXCR3 chemokine receptors, whereas Th2 lymphocytes express CCR4, CCR8, and CCR3 to a lesser extent (47, 56).

**Figure 1:**
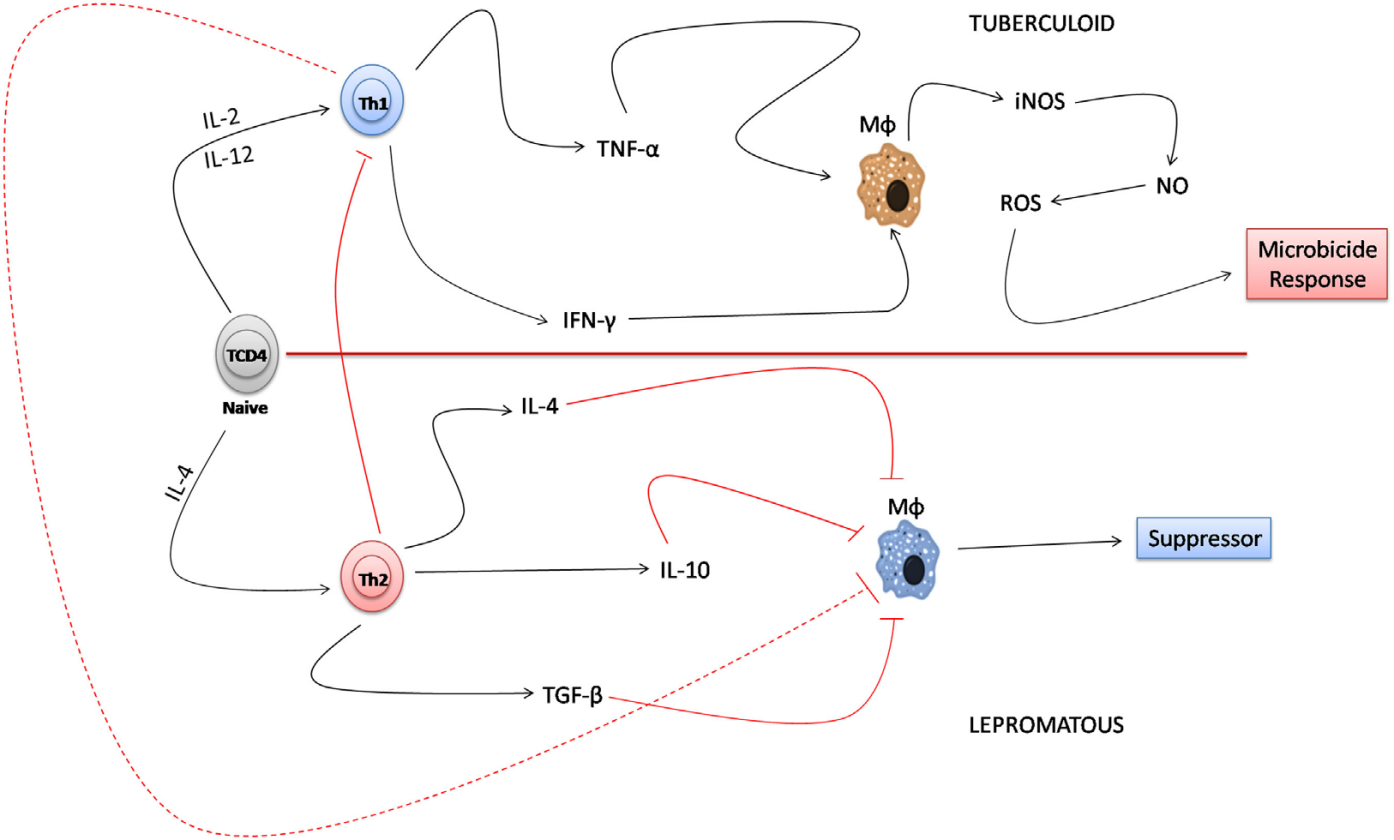
Immune paradigm of the T helper (Th) 1/Th2 response in the polar forms of leprosy. In the tuberculoid form, Th1 lymphocytes, when producing tumor necrosis factor-alpha (TNF-α) and interferon-gamma (IFN-γ), induce the activation of macrophages that produce induced nitric oxide synthase (iNOS) and NO that destroy *Mycobacterium leprae*. In the lepromatous form, the predominance of the Th2 lymphocyte response to Th1 detriment results in the inactivation of the microbicidal response of macrophages mainly due to the response of cytokines, such as IL-4, IL-10, and TGF-β, that favor the survival of *M. leprae*.

In the immunological paradigm of the interaction of Th1 and Th2 that was established many years ago, the tuberculoid form represents the clinical form characterized by a smaller number of bacilli with a granulomatous infiltrate composed of macrophages and lymphocytes. Concomitant to the immune response, in the resistant form of the disease, the decrease in bacillary load is associated with a Th1 response pattern where the production of TNF-α and IFN-γ activate macrophages and induce the production of iNOS that destroys the bacillus due to the release of free radicals. The lepromatous form, which is considered the susceptible form of the disease, is associated with a greater number of lesions with the presence of foamy macrophages and globes. According to the literature, there is a predominance of a Th2 lymphocytes response in the lepromatous form, which induces the production of cytokines such as IL-4, IL-10, and TGF-β that inactivate the microbicidal response of macrophages, thereby facilitating the survival of the bacillus (57–61). In this constructed environment, the Th2 lymphocyte-mediated response releases IL-4, IL-10, and TGF-β which negatively regulates the Th1 response by inhibiting the microbicidal response of macrophages in the susceptible pole of the disease (56, 61) (Figure 1).

## Identification of New Subpopulations of T Lymphocytes in Leprosy

With the advancement of general knowledge of immunology, the interpretation of the dual response paradigm between the Th1 and Th2 lymphocytes in the tuberculoid and lepromatous polar forms has been reconsidered with respect to incorporation of new mechanisms by which T cells can develop to contribute to the immunopathogenesis of the disease. Recent studies have shown that through the differentiation of T CD4+ lymphocytes between the tuberculoid and lepromatous polar forms, Th9, Th17, Th22, and Treg lymphocytes induce mechanisms that go beyond the standard established by the classical Th1 and Th2 paradigm described above (6–8, 47, 48).

### Th9 Lymphocytes

In the Th9 lymphocyte response pathways, early reports demonstrated that the cells arise from Th0 lymphocyte differentiation by which IL-4 and TGF-β change the behavior of the “naive” CD4+ cells and transform them into Th9 lymphocytes; the main transcription factors involved in the process are STAT6, IRF-4, and PU.1 (62–65). In the polar forms of the disease, Th9 lymphocytes produce cytokines such as IL-9 and IL-10 that alter the pattern of the immune response, whereas in the tuberculoid form, IL-9 induces the development of the microbicidal response of macrophages through synergism of the response among IFN-γ, IL-6, and IL-12 (66, 67). In addition, in the tuberculoid form, IL-9 negatively regulates the production of IL-10, showing that IL-9 has a proinflammatory effect (67). In the lepromatous form, the response of Th9 lymphocytes presents a distinct behavior by which IL-9 inhibits the production of IL-4 as well as IFN-γ and TNF-α. In contrast, IL-10 contributes to the development of the immunosuppressive response by inactivating the microbicidal response of macrophages *via* positively regulating the production of TGF-β (67).

### Th17 Lymphocytes

T helper 17 lymphocytes are part of the group of T lymphocyte subtypes that, like Th1, produce proinflammatory cytokines, with IL-17 as the main representative. In leprosy, the main cytokines that participate in the cell differentiation process are IL-6 and TGF-β, and the main transcription factor involved is RORγ (68–70). These cells are characterized by the expression of the surface markers CD161, IL-23R, CCR6, and CCR4 (68, 69). Because this cellular subtype has proinflammatory activity, the response of Th17 lymphocytes has been reported to be associated with inflammatory processes consistent with reverse reactions (68–71). An increase in IL-17 expression has been found in the tuberculoid form, and this cytokine elevation contributes to the recruitment of inflammatory cells, activation of endothelial cells, and maintenance of the chronic inflammatory process (71, 72). The response dynamics of Th17 lymphocytes have been suggested to be crucial for modulation of macrophage activity, since in the tuberculoid form, IL-17 induces the production of TNF-α, IL-6, and iNOS, leading to the production of reactive oxygen intermediates that destroy the bacillus (73, 74). In the resistant form of the disease, owing to the toxic environment generated, IL-17 potentiates the pro-inflammatory response. At the level of neural involvement, the Th17 lymphocyte response has been associated with an inflammatory demyelination process caused by tissue damage. In this context, when the inflammatory process is established, IL-17 negatively regulates the production of nerve growth factor (NGF) and its receptor in both the tuberculoid and lepromatous forms, thus contributing to the appearance of more severe neural lesions (74).

### Th22 Lymphocytes

T helper 22 lymphocytes have recently become recognized as an important subpopulation involved in the immune response to infectious agents. These cells are part of a group of T CD4+ cells that secrete FGF family isoforms and cytokines such as IL-22, TNF-α, IL-13, and IL-26; however, as a peculiarity, they do not produce IL-17 or IFN-γ. These cells have chemokine receptors such as CCR4, CCR6, and CCR10, and undergo differentiation in the presence of IL-6 and TNF-α (75–80). Early reports on leprosy showed increased expression levels of IL-22, IL-13, and FGF b in the lepromatous form, whereas an increase of TNF-α was more evident in the tuberculoid form. Among the cytokines that compose the response profile of the cell, IL-22 has been particularly highlighted in leprosy owing to the fact that in the lepromatous form, this cytokine participates in the mechanisms of maturation of the phagolysosome. Indeed, in macrophages infected by the bacillus, IL-22 was shown to modulate the production of calgranulin A, thereby increasing the intracellular concentrations of Ca^2+^ as well as Rab7 (81). As a detriment to the development of a macrophage response, IL-22 induces the production of STAT3 as well as iNOS that destroy the bacillus (82). *In situ*, the Th22 lymphocyte response has gained fundamental importance in the lepromatous form of the disease, because FGF b can regulate different cellular functions that can interfere with the processes of cicatrization, migration, cell division, proliferation, differentiation, and angiogenesis. In these circumstances, the increase of FGF b in the lepromatous form of the disease reinforces the crucial role of this growth factor in development of the reparative response, since this clinical form is associated with greater bacillary spread, greater tissue damage, and, consequently, a greater number of injuries (11, 81).

### Regulatory T Lymphocytes

Treg lymphocytes represent another subpopulation of lymphocytes with the phenotype CD4+ CD25+ FoxP3+, which are also involved in the immunopathological response in leprosy. Treg cells express on their surface the chemokine CXCR4 and CCR5 molecules (83–86). TGF-β and IL-10 are the main cytokines involved in cell differentiation, under control of the regulatory transcription factor FoxP3. In leprosy, Treg cells have been described as the cells that maintain the balance of the response between Th1 and Th2 lymphocytes. The increase of FoxP3 has been reported in the lepromatous form of the disease, which is associated with the anti-inflammatory nature of this cell type (83–86). In reactional forms, the expression of FoxP3 has been detected in erythema nodosum leprosum along with granulomatous infiltration (87).

The presence of FoxP3 has been shown to be crucial for development of the apoptosis response, which positively regulates caspase-3 production in both the tuberculoid and lepromatous forms (88). In the development of the immunosuppressive response, Treg cells producing TGF-β and IL-10 contribute to inhibition of the production of proinflammatory cytokines as well as to the development of the Th1 and Th17 lymphocyte response in the lepromatous form of the disease (85–88). Within the generalized response paradigm of helper T cells in the lepromatous form, Tregs play an important role in the induction and maintenance of the immunosuppressive response that contributes to the survival of the bacillus in the lesions (89).

## New Cytokine Profiles and Specificities in the Clinical Evolution of Leprosy: An Overview

Leprosy is a chronic infectious disease whose clinical course is classically linked to the immune response pattern where the clinical and evolutionary aspects of the infection depend on the relationship between the infectious agent and the immune response of the host (6–8). Based on this pathogen-host relationship, criteria guiding clinical classifications that were developed depending on the pathogenesis of the disease and widely used in several studies were defined. In this context, Ridley and Joplin in 1966 (5) described the clinical forms of the disease, and later conducted further studies, defining the pattern of cytokine expression in each of the forms described by the authors (6). These studies formed the basis for other works on immunology in infectious diseases, making leprosy an excellent model for investigating the pathogen–host relationship (36–38). With the advancement in the knowledge about cellular and humoral immunology, new populations of lymphocytes and macrophages (M2, M4, and M17) were identified, suggesting great complexity of the immune response in infectious and inflammatory processes in general, and opening an extensive field of research, further increasing the challenge to study these processes (11, 90–96). Considering the cytokine profiles, the emergence of new cellular subpopulations, such as Treg, Th9, Th22, and Th17, and their relation with the immunopathogenesis of leprosy, increased the challenge for investigation of this complex disease; on the contrary, it opened horizons for understanding new processes, which, based on the duality of the Th1 and Th2 paradigm, could not be studied more extensively until then (65, 67, 75–77, 79, 89). Therefore, although the mechanisms of the whole process became more complex, they resolved multiple questions that were still obscure, facilitating the understanding of the evolution of *M. leprae* infection.

Importantly, many of these profiles act in an integrated way, contributing multiple times to the adequate control of the immune response and induction of a regenerating environment that can prevent or even delay the occurrence of neural lesions and deformities, which are one of the cadres that induce permanent disability in these patients (65, 67, 75–77, 79, 89).

The understanding of the pathogenesis of the neural lesion reveals that regardless of the way (nerve endings with retrograde axonal flow, phagocytosis by the perineural cells, or the endoneural vessels) the bacillus reaches the nerves or Schwann cells, the presence of *M. leprae* in the endoneural macrophages and the rupture of the Schwann cells by bacillary replication triggers a perineural inflammatory response that contributes to myelin destruction and consequent neural damage (10, 97, 98). On the contrary, in an attempt of the host to prevent the evolution of neural lesions and deformities, some of the cytokines present in the Th17 or Th22 profiles, such as TGF-β and FGF b, may induce neural regeneration or be associated in the induction of NGF production that is capable of inducing neural regeneration (56, 61, 70). The role of new cytokine profiles in the pathogenesis of neural involvement has not yet been adequately evaluated and studies in this field are not available in literature; however, it is evident that several cytokines act on these new profiles as they can control the intensity of the host immune response or induce a regenerative environment, which may be a determinant in the evolution of the lesion (56, 61, 65, 67, 70, 75–77, 79, 89).

Another important aspect to consider is the role of these new lymphocytic profiles in the dimorphic or borderline forms of leprosy, as well as their role in the immunopathology of leprosy reactions (6, 23). Several cytokines that are characteristic of these profiles also play an important role in determining the intermediate and reactional forms (65, 67, 75–77, 79, 89). Regarding the classical Th1 and Th2 duality, depending on the clinical form considered, the predominant profile may assume patterns with increased levels of pro- or anti-inflammatory cytokines or mixed patterns, as well as the presence of Treg lymphocytes may be more frequent in the borderline-lepromatous and lepromatous leprosy (6–8, 47, 48). Some studies have suggested an important role of cytokines, such as IL-17, in tuberculoid leprosy and reactive forms of the disease, but the increase in this cytokine has not been reported as a predictive factor for leprosy reactions (68–74). Th1 profile cytokines, such as IL-2, INF-γ, and TNF-α, which promote differentiation of naïve T lymphocytes and macrophages, as well as the cytokines—TGF-β, IL-17, and IL-23—and Treg lymphocytes are involved in the pathogenesis of the type I reaction or reverse reaction (6, 23). Profiles, such as Th9, due to their role in tuberculoid leprosy, and the characteristic expression of IL-9, that shows synergistic biological actions with IFN-γ and IL-12, reveal its important role in the immunopathology of the type I reaction, but these data need to be confirmed by specific studies (65, 67). Cytokines, such as IL-1β, IL-4, IL-6, and TNF-α, are key components in the pathogenesis of type II reaction or erythema nodosum. Profiles, such as Th22, which have the characteristic of producing TNF-α and IL-6, without IL-17, may contribute to the evolution and pathogenesis of the type II reaction that, together with the Th2 profile, is associated with the development of lepromatous leprosy, where the neural damage is more intense and there is need for a more evident regenerative environment (75–77, 79, 81).

Finally, the advancement in immunology, in particular, the immunology of infectious diseases, and more so, the immunology of leprosy, while increasing our knowledge about the complexity of the immunopathogenesis of the disease, opens up several research opportunities for better understanding and development of therapeutic targets in leprosy (99, 100, 101) and infectious diseases, as classically already reported in leprosy immunology studies.

## Conclusion

**Figure 2:**
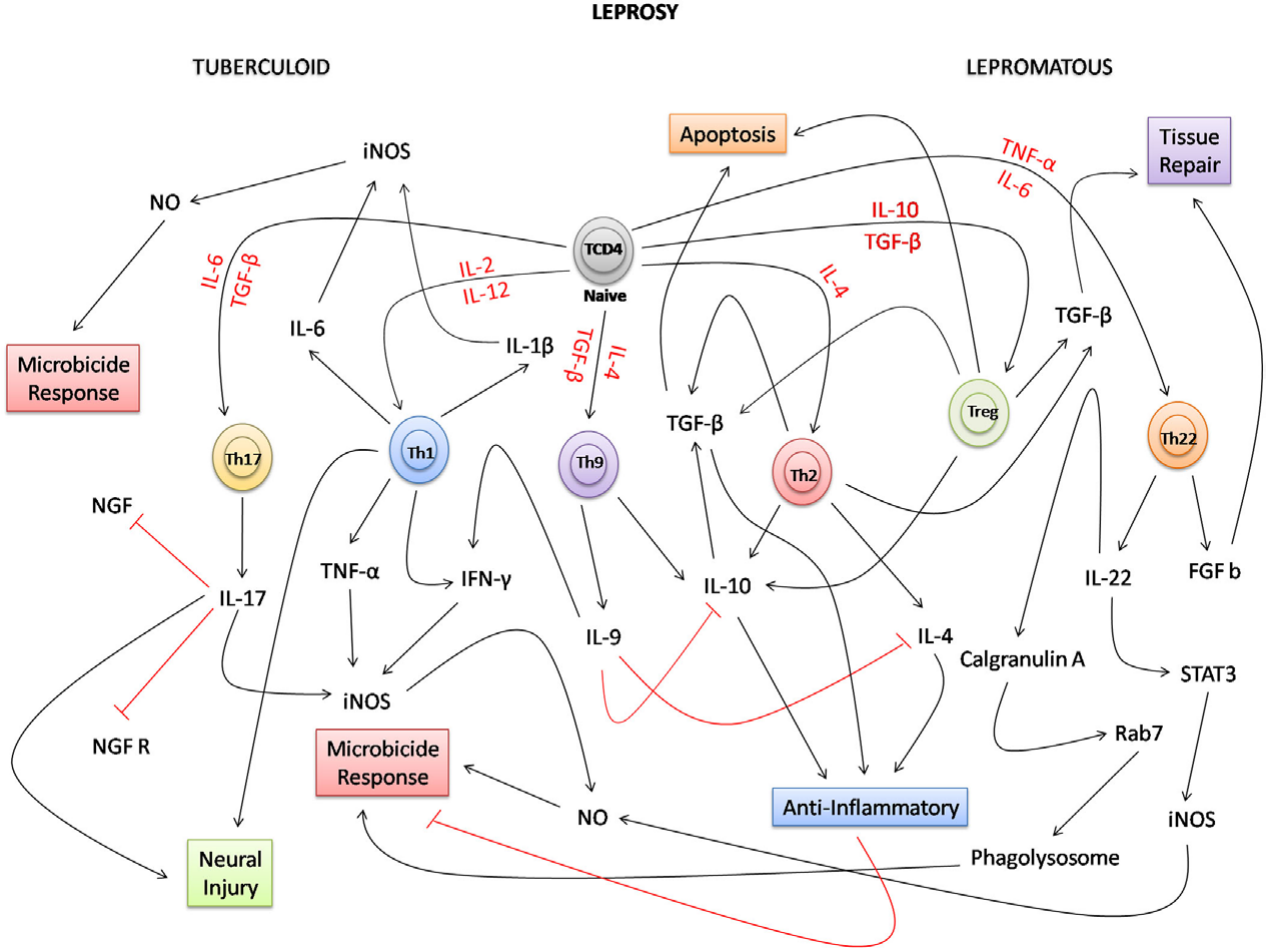
Possible network integration and new immune paradigms in the response of T lymphocytes in leprosy. Due to the complexity of the immune response, in the polar forms of leprosy, naive TCD4 lymphocytes under the influence of several cytokines (red) can differentiate into several T subpopulations [T helper (Th) 1, Th2, Th9, Th17, Th22, and Treg]. In the tuberculoid form, Th1, Th9, and Th17 lymphocytes participate directly in the proinflammatory response, inducing the production of cytokines, such as IL-1β, IL-6, IL-9, IL-17, tumor necrosis factor-alpha (TNF-α), and interferon-gamma (IFN-γ), and the microbicidal activity of macrophages. Because of the intensity of the inflammatory process, IL-17 negatively regulates the production of nerve growth factor (NGF) and the NGF receptor (NGFR), potentiating the neural damage together with the Th1 lymphocyte response. In the lepromatous form, Th2 and Treg lymphocytes participate in the anti-inflammatory response producing IL-4, IL-10, and TGF-β cytokines that inhibit the activation of macrophages, facilitating the survival of *Mycobacterium leprae*. In this clinical form, apoptosis has a strong relationship with the performance of Treg cells and TGF-β. In the Th9 response, IL-9 downregulates the production of IL-4 and IL-10. In the clinical form, where the number of lesions is much higher than that in the tuberculoid form, the performance of growth factors, such as TGF-β and Basic fibroblast growth factor (FGF b), is fundamental to induce tissue repair. Considering the response of Th22 lymphocytes, new approaches have shown that, in the bacillus-infected macrophages, IL-22 positively regulates the production of calgranulin A and STAT3. This interferes not only in the process of maturation of the phagolysosome due to the expression of Rab7, but also in the microbicidal response of macrophages where STAT3 induces the production of induced nitric oxide synthase (iNOS), and consequently NO, that destroys *M. leprae*.

Overall, this review highlights the advances in the field of immunology that have been gained in recent years. Given the complexity of the mechanisms by which T CD4+ lymphocyte subtypes develop, it is evident that although the classical immunological paradigm on the interaction between Th1 and Th2 lymphocytes is still relevant, the identification of new actors (Th9, Th17, Th22, and Treg) expands the interpretation of the immune response in the disease spectrum. This paradigm should be reconsidered mainly due to the appearance of new cytokines that modulate the patterns of activation and development of the response of macrophages in the clinical spectrum of leprosy (Figure 2). In this context, future perspectives based on this new knowledge can help to broaden the immunological discussion related to the response of macrophages, how they differ, how important they are, and how the new subpopulations that have emerged (M2, M4, and M17) may be involved in the immune response against *M. leprae*. In addition, in the T lymphocyte response pathways, further research should focus on understanding how Th9, Th17, Th22, Treg, and a new T lymphocyte subpopulation known as Th25 participate in establishment of the complex immune response against *M. leprae*.

## Author Contributions

JS, MS, and JQ conceived and wrote the manuscript.

## Conflict of Interest Statement

The author declares that the research was conducted in the absence of any commercial or financial relationships that could be construed as a potential conflict of interest.
